# Solid PRF Serves as Basis for Guided Open Wound Healing of the Ridge after Tooth Extraction by Accelerating the Wound Healing Time Course—A Prospective Parallel Arm Randomized Controlled Single Blind Trial

**DOI:** 10.3390/bioengineering9110661

**Published:** 2022-11-08

**Authors:** Shahram Ghanaati, Joanna Śmieszek-Wilczewska, Sarah Al-Maawi, Pauline Neff, Homayoun H. Zadeh, Robert Sader, Anja Heselich, James L. Rutkowski

**Affiliations:** 1 FORM-Lab, Department for Oral, Cranio-Maxillofacial and Facial Plastic Surgery, Medical Center of the Goethe University Frankfurt, Goethe University, 60590 Frankfurt am Main, Germany; 2 Department of Dental Surgery, Faculty of Medical Sciences in Zabrze, Medical University of Silesia, 40-027 Katowice, Poland; 3 VISTA Institute for Therapeutic Innovations, Woodland Hills, CA 91367, USA; 4 Restorative Dentistry, School of Dental Medicine, State University of New York, Buffalo, NY 14214, USA

**Keywords:** PRF, socket seal, ridge sealing, LSCC, RCT, guided open healing

## Abstract

Systematic evaluations regarding the influence of PRF in ridge sealing are still lacking. To the best of our knowledge, this is the first systemic randomized, controlled, clinical approach dealing with the potential of a systematic applied solid PRF on soft tissue socket healing of molar and premolar extraction sockets with evaluation for up to 90 days. Qualitative and quantitative image analysis showed that PRF contributed to a significantly faster ridge sealing, within the period of 7–10 days in both tooth types. This led to a visibly less contraction at the PRF-treated group sites at day 90. Patients’ pain perception demonstrated no statistic significance between both groups (PRF vs. natural healing), but the patients in PRF group seemed to have had less pain throughout the observational period. It becomes evident that PRF is able to serve as a promotor of the secondary wound healing cascade. The guiding capacity of PRF accelerating the process of open ridge healing makes it possible to act as a natural growth factor drug delivery system, providing a more predictable guided open wound healing of the ridge with less contraction of the soft tissue, the latter being a key factor for the subsequent successful dental implantation and oral rehabilitation.

## 1. Introduction

When it comes to the functional and esthetic replacement of lost teeth, dental implants represent the most comfortable and favorable solution for the patients [1,2]. For this option to succeed in the long term, different clinical, biomechanical, and biological requirements have to be met [3,4], to allow for an adequate osseointegration of the dental implants. One crucial point is to fully understand the process of socket healing and its implications on dental implantology. For this reason, in the last 25 years, these mechanisms have been the focal point of multiple studies within the corresponding field of research [5,6]. It was demonstrated that tooth loss results in a remodeling process of the alveolar bone that negatively affects bone quantity and quality [7], which leads to continual bone atrophy. Further investigation characterized this process as a fast-paced and continuous phenomenon that results in the loss of 50–60% of the alveolar bone three months following tooth extraction [5,8].

Within the last ten years, multiple fields of regenerative medicine have been increasingly appreciative of blood concentrate systems [9]. Blood concentrates are derived from the patient’s peripheral blood [10]. After collection of a patient’s whole-blood, centrifugation is used to concentrate the relevant components, e.g., leukocytes, platelets, growth factors and plasma proteins. In this context, a variety of centrifugation and processing protocols [11,12,13] have been applied, posing a challenge for comparison of the multiple techniques. The so-called platelet-rich plasma (PRP) represents the first generation of blood concentrates. It consists mainly of platelets and is obtained via two separate centrifugation steps with a rather high relative centrifugal force (RCF) [11,14]. Leukocytes are removed during the second centrifugation step for PRP production [11,15]. Another blood concentrate that is obtained by using high RCF is the plasma rich in growth factors (PRGF). As the name suggests, this blood concentrate capitalizes on the advantages of blood-derived growth factors [16].

A one-step centrifugation without the addition of anticoagulants characterizes the production process of the second generation of blood concentrates [17]. This includes the well-known platelet-rich fibrin (PRF) that contains platelets and leukocytes as well as the corresponding subgroups embedded in a fibrin matrix with plasma proteins [9]. Its earliest version, the Leukocyte-rich Platelet-Rich Fibrin (L-PRF), was based on a centrifugation process that applied a lower 710x*g* RCF than its predecessors [18]. However, 710x*g* can still be considered a high RCF. A systematic reduced RCF at a constant centrifugation time of 8 min has demonstrated that the low speed centrifugation concept (LSCC) leads to an increased number of blood inflammatory cells and the growth factors contained within them [19].

This brief history of blood concentrates highlights a key problem: within a relatively short period of time, multiple protocols for obtaining blood concentrates have not only been introduced, but also formed the basis for additional scientific investigation within the field. Thus, it is extremely difficult to adequately compare the research findings of one technique with another [20]. Moreover, some publications do not provide sufficient information to confirm the preparation steps. This has allowed for marketing to dictate what has become popular vs. what true scientific research would support.

Nevertheless, recent literature addressing soft tissue healing and postoperative pain in dental extraction sockets treated with or without PRF blood concentrates reveals significantly better and faster wound healing, and significantly less postoperative pain when sockets are filled with PRF [21,22,23,24,25,26]. Other studies did not observe significant changes between treatment with or without PRF; however, they did reveal at least a tendency for faster and better soft tissue healing [26,27,28] and less postoperative pain [14,27,29]. However, there are varying protocols used for the PRF preparation described in the studies, e.g., L-PRF [22,23,26,27], A-PRF+ [27], with little or no specific information regarding the preparation protocols. This makes it difficult to compare the findings.

In order to reduce data heterogeneity in the future, it is suggested to standardize the various protocols including documentation of the RCF. Against this background, the aim of the present randomized controlled clinical trial was to explore the effects of solid platelet-rich fibrin on the: (i) dynamics of soft tissue related ridge sealing, and (ii) patients’ subjective pain perception after tooth extraction in comparison to spontaneous healing within a time course of 90 days in premolars and molars separately. In the present study, solid PRF with a RCF of 177x*g* was used, for which it has been demonstrated to have significantly more cells and growth factors, as was shown for RCF of 710x*g*, when keeping the time constant [19]. By comparing the processes involved in ridge sealing triggered by this solid PRF protocol and that of a control group not receiving PRF treatment. To the best of our knowledge, this study is the first to analyze the effect of PRF on the process of soft tissue wound healing on a ridge after tooth extraction in premolars and molars in comparison to the control group. At the same time, this study focuses on pain perception in such a systematic approach, with a long-term observation period of soft tissue healing of up to 3 months. Both are aspects of great clinical interest, especially as the perceived pain might be associated with the time course the ridge needs to be sealed.

Knowledge about PRF-related ridge sealing capacities and patient’s pain perception will have a high clinical impact on the necessity of PRF application following tooth extractions.

## 2. Materials and Methods

### 2.1. Study Design

This prospective, parallel-arm, randomized controlled clinical trial (RCT) was conducted at the Medical University of Silesia in Katowice, Poland between January 2018 and May 2022. The study adhered to the principles described in the Declaration of Helsinki and the national regulations of Poland and Germany concerning human-based studies. Surgical interventions and follow-up documentation, performed by the authors SG and JS, were covered by the ethical approval given by the Silesian Medical Council, Katowice, Poland (#30/2017). Following tooth extraction(s) and either treatment with solid PRF or no treatment, primary closure was not achieved or desired. The intent of the study was to access the healing of the open wound (secondary intention wound healing). All participants were informed of the study’s procedures and objectives. Participants were included only after providing written informed consent.

#### 2.1.1. Inclusion and Exclusion Criteria

Patients ≧ 18 years of age requiring premolar or molar tooth extraction(s) in the mandible or maxilla region (except for the 3rd molar extraction), and who were planned for dental implant therapy and restoration, were recruited. Complete medical and dental histories for each patient were obtained. Excluded were patients without written informed consent, pregnant women, patients with decompensated metabolic diseases, patients with periodontal disease without successful previous treatment, patients with periapical lesions, or patients with a risk for MRONJ due to bisphosphonate treatment. Further exclusions were patients with impaired oral hygiene or who were unable to follow the necessary-study related instructions. No cases with root fractures and with risk for residual fragments were included. No cases with alveolar bone defects pre-extraction were included.

#### 2.1.2. Sample Size Calculation

In accordance with our statistical institute, the sample size calculation was determined and based upon criteria described elsewhere [22]. A sample size of at least 13 extractions per group were required to achieve an 80% power at a significance level of 5%. According to experience, a drop-out rate of 20% was estimated, leading to a sample size of 16 extractions per recruited group.

#### 2.1.3. Randomization

Patients were randomly allocated to either the control group or to the socket preservation treatment group with solid PRF. Based on sample size calculation, computer-based randomization using the calculation tool by GraphPad (GraphPad Software, LLC., San Diego, CA, USA) was created by a study member not involved in the patients’ treatment. For each planned patient, a randomization letter was prepared in a sealed envelope, which was opened by the surgeon directly before tooth extraction.

#### 2.1.4. Follow-Up

Tooth extraction and treatment according to the allocated group were clinically observed and evaluated for wound healing at day 1-, 3–5-,7–10-, 90-days post extraction. Implantation was planned 3 months post extraction followed by the final prosthetics within 3–5 months of post-implantation.

#### 2.1.5. Outcome Measures

The primary outcome was the evaluation of soft tissue healing kinetics in post extraction sockets between the test and control group. The secondary outcome measures included pain perception, and implant stability and survival.

### 2.2. PRF Preparation

Autologous platelet rich fibrin (PRF) was prepared, as previously described elsewhere [9,19,30]. For patients within the experimental PRF treatment group, the patient’s peripheral blood was collected in special PRF blood vacuum 10 mL red-top glass sampling tube(s) without additives (Process for PRF, Nice, France) for eventual centrifugation, processing, and application at the selected premolar and molar extraction sites. For each extracted tooth, one glass tube was collected per root. The decision for the amount of the tubes was made based on the X-ray analysis of the tooth prior to extraction and after randomization.

Filled sampling tubes were centrifuged in a PRF-Duo Quattro medical device centrifuge for PRF (Process for PRF, Nice, France). If needed, a glass tube was filled with physiological sodium chloride and used as a balancing counterpart during centrifugation. All tubes were centrifuged within 3 min of collection.

Solid PRF was gained at 1200 rpm (177x*g*) for 8 min. It was harvested by carefully removing the coagulated PRF clot using sterile tweezers. The attached residual red blood cell phase was carefully scraped off with the blunt side of sterile scissors and the remaining solid PRF clot was transferred to a PRF processing box (Process for PRF, Nice, France) for pressing into a solid PRF plug. The PRF clot was placed into the designated cavity within the processing box and pressed by application of the special weight cover and gravitational force without the further uncontrolled application of additional manual pressure. The resulting cylindrical solid PRF plug was applied into the extraction socket within 20 min upon removal from the processing box.

### 2.3. Surgical Procedures

After clinical examination, patients underwent orthopantomography (OPG) or computed tomography (if indicated) to verify the need for dental extraction(s). Periodontal treatments, including supra and subgingival scaling in conjunction with plaque removal were provided, and further supported with proper oral hygiene instructions.

#### 2.3.1. Tooth Extraction and Socket Preservation

Tooth extraction(s) were performed under local anesthesia using a simple minimal traumatic technique. Vertical releasing incisions were omitted. To minimize the risk for root and bony fractures, molar teeth were sectioned via a piezo technique. After exodontia, a rigorous inspection and curettage of the socket was performed, followed by rinsing with a sterile 0.9% saline solution. Premolar or molar extraction sockets allocated to the PRF treatment group were filled with solid PRF. Filled extraction sockets were sutured with tension free non-resorbable sutures in a horizontal mattress manner. Extraction sockets allocated to the control group were left untreated but had a similar suture applied. Primary closure was not achieved nor desired. The intent of the study was to access the healing of the open wound (secondary wound healing).

#### 2.3.2. Follow-Up

Photo documentation of wound healing, pain perception via visual analogue scale, analgesic consumption as well as clinical observation of adverse events were performed at days 1, 3–5, and 7–10 post extraction. Suture were removed at the day 7–10 visit. Additionally, prior to implantation 3 months post extraction, photo documentation of soft tissue healing and a CBCT evaluation of bone regeneration were obtained.

### 2.4. Clinical Measurements

#### 2.4.1. Socket Closure Evaluation

Wound healing assessment of socket sealing was performed using image analysis software for photo documentation following the tooth extraction at all evaluation time-points. Image analysis software used was *Fiji Image J* Image Analysis Software (imagej.nih.gov, NIH, Bethesda, MD, 20892, USA [31]; last accessed on 1 October 2022). The scale was set using a reproducible reference (e.g., periodontal probe) for each time point (immediate sutured socket, days 1, 3–5, and 7–10 post-extraction). A polygonal tool was used to define the wound area as the Region Of Interest (ROI) and the wound size area was measured within the tool. The wound area was defined as follows: the initial wound rim of the extraction socket defined the wound area and was marked using the polygonal tool. In the follow-up documentation, the transition from non-epithelialized tissue and epithelialized tissue defined the wound border. Calibration of the analysis was conducted by definition of the wound border criteria by the experienced maxilla-oral-facial surgeon and oral surgeon. The marking of the wound border and area for analysis by an analyst was confirmed by both experts, and the blinded cross-evaluation was performed to confirm reproducible results before the analysis of the study data was conducted. The wound area definition and evaluation was further checked and verified on a random basis by experts.

For evaluation, the initial wound post extraction was defined as 100%. The ridge sealing progression was calculated as a residual wound size relative to the initial wound. Patients with photo documentation lacking suitable reference for scale or where ROI could not be defined for each time point due to artefacts were excluded from evaluation.

#### 2.4.2. Pain

Pain perception was evaluated at post-op days 1, 4, and 7 via visual analogue scale (VAS) ranging from 0–10, 0 defining no pain, and 10 defining the worst pain possible. Included in the evaluation were only those patients providing consistent documentation at all post extraction time points.

### 2.5. Statistics

**Figure 1:**
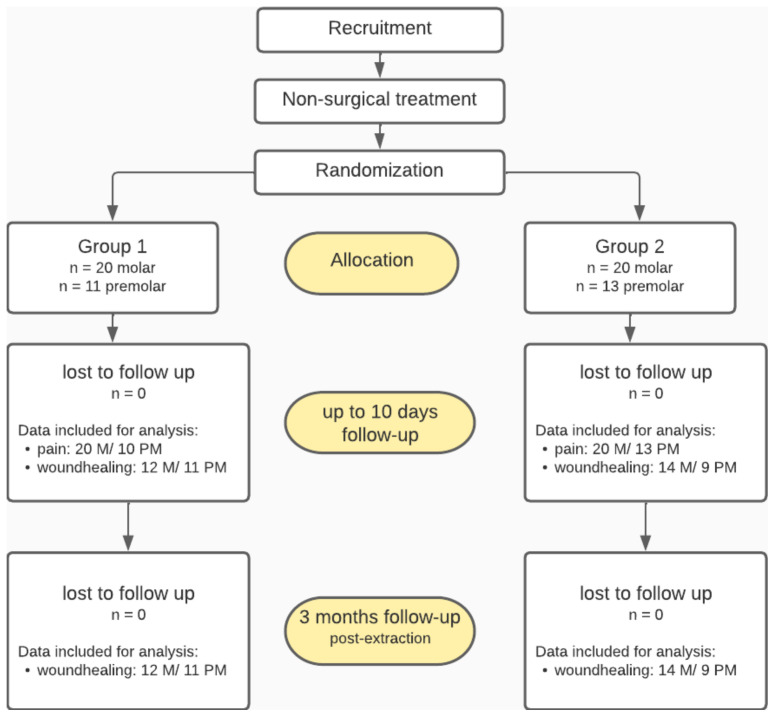
**Study design flow chart.** Study design, recruitment, loss to follow up, and data sets evaluated.

The descriptive statistics and statistical analysis were calculated using GraphPad Prism Analysis software (version 9.3.1, GraphPad Software, LLC., San Diego, CA, USA). For case distribution and size of data sets per group analyzed for the single endpoints see Figure 1.

The statistical analysis of pain (VAS) was conducted via the Two-way ANOVA (molar, n_(ctrl)_ = 20, n_(solidPRF)_ = 20) or Mixed-effect analysis (premolar, n_(ctrl)_ = 10, n_(solidPRF)_ = 13), both with Tukey’s multiple comparisons post hoc test.

The statistical analysis of wound healing and the respective residual socket size at day 7–10, was conducted by calculating the mean values normalized to the baseline wound size of the respective wound size after suture. Statistical differences between groups were analyzed via the unpaired two-tailed *t*-test (molar, n_(ctrl)_ = 12, n_(solidPRF)_ = 14) (premolar; n_(ctrl)_ = 11, n_(solidPRF)_ = 9) with a CI of 95%.

## 3. Results

In this study, early as well as late dynamics of ridge sealing following tooth extraction with intentional open-wound healing (secondary wound healing) were analyzed within a time period of up to 90 days in both the experimental as well as the control groups. The assessment of identical digital images, which were captured within the same perspective at each of the investigation time points, allowed for the progressive evaluation of the wound healing process. The boney defect with a resultant soft tissue wound was initiated as a result of a tooth extraction. The healing evaluation aimed to evaluate the progressive soft tissue closure (or coverage) of the resulting bony defect, i.e., the so-called socket inside the alveolar ridge. Based on the present study design, the healing dynamics during the secondary wound healing process was observed in premolar and molar extraction sites. The analyses were performed qualitatively as well as quantitatively, as described in the Materials and Methods section. The data for premolars and molars will be presented for each of the study groups independently.

### 3.1. Qualitative Assessment of Socket Sealing Process within the Control Group

Qualitative analysis of the sealing process within the control group will be presented individually for both premolar and molar non-treated extractions.

#### 3.1.1. Socket Sealing in Premolars

**Figure 2:**
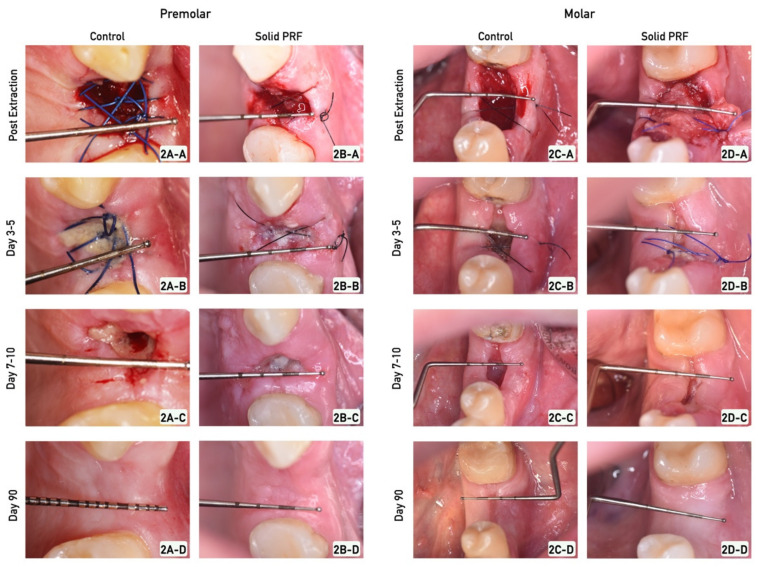
**Wound healing progression.** Representative images of wound healing progression post tooth extraction and at days 3–5, 7–10, and 90. Left panel represents representative cases for premolars (control group (**2A**), solid PRF group (**2B**)), and right panel for molars (control group (**2C**), solid PRF group (**2D**)).

In the non-treated (control) group, tooth extraction and the post-operative course were uneventful (Figure 2A).

Within the time prior to 7–10 days, the sealing process within the premolar group resulted in a cone-like connective soft tissue coverage of the socket (Figure 2A). Between days 3–5 after extraction, coverage of the socket with epithelium seemed to have initiated from the buccal and lingual flap edges towards the center of the wound (Figure 2A-B). Between days 7–10 after extraction, the base (ground) of the wound was still not covered. Since the wound was still open, this indicates that the epithelium had not healed or reached the bottom (apical portion) of the wound (Figure 2A-C). Accordingly, no complete ridge sealing was reached within the observed early time points of the study (Figure 2A-C). At day 90, the ridge was completely and homogenously re-epithelialized (Figure 2A-D). The epithelium, which covered the ridge, appeared as a whitish epithelium layer. Thus, the new built tissue on the ridge resembled scar tissue, which seemed to be under tension resulting in contraction (Figure 2A-D). It seemed as if the contraction had contributed to the loss of the buccal and lingual width aspect of the former socket walls by pushing them towards each other, in order to enable the closure of the wound (Figure 2A-D).

#### 3.1.2. Socket Sealing in Molars

In this control (non-PRF treated) group, the tooth extraction and the post-operative course were also uneventful (Figure 2C-A). The sealing process in molars within the control group demonstrated only to some extent the similarities to the process of ridge sealing in premolars (Figure 2C). Within 3–5 days after tooth extraction, the wound base (ground) for the molars was clearly visible below the level of the buccal and lingual soft tissue flap edges (Figure 2C-B). However, at 7–10 days after tooth extraction, the socket seemed to be completely re-epithelialized (Figure 2C-C) but not filled. These findings are in contrast to those in premolars of the same group (Figure 2A-C). The epithelization seemed to have led to less expression of scar-like tissue (Figure 2C-C). Accordingly, the soft tissue seemed not to be under as much tension and thus contraction, when compared to the findings in comparative groups for premolars (Figure 2C-C). At day 90, the buccal and lingual aspects of the former sockets seemed to be less contracted towards each other, when compared to the findings observed for premolars of the same time period group (Figure 2C-D). At this time point, the level of the flap edges seemed to have been equalized with that of the wound base (ground) (Figure 2C-D).

### 3.2. Qualitative Assessment of Socket Sealing Process within the Experimental Group

In the following, the qualitative analysis of the sealing process within the experimental (PRF treated) group will be presented for premolars and molars individually.

#### 3.2.1. Socket Sealing in Premolars

In this treatment group, tooth extraction and the post-operative course were also uneventful (Figure 2B-A).

Within 3–5 days, the sealing process within the solid PRF group, i.e., tooth extraction and socket augmentation with solid PRF implantation, resulted in a faster reduction of the outer wound diameter on the ridge when compared to the findings in premolars of the control group (Figure 2B-B). It seemed as if the flap edges used the solid fibrin as a basis to horizontally seal the ridge within the first 7–10 days following tooth extraction. The PRF seemed to guide ridge sealing (Figure 2B-C). Thus, no unepithelialized wound was detectable at the base (ground) with this group in the time course of 7–10 days post-operatively (Figure 2B-C). Accordingly, no cone-like shape of the former socket was detectable when observing the re-epithelialization process (Figure 2B-D). Almost no contracture was detected during the ridge sealing process until day 90 after extraction (Figure 2B-D). At day 90 in this treatment group, the ridge was completely and homogenously re-epithelialized (Figure 2B-D). The soft tissue did not appear to be under tension or have resultant contraction (Figure 2B-D). Consequently, almost no loss of the buccal to lingual width of the former socket walls could be observed, resulting in a ridge shape with a prominent horizontal dimension (Figure 2B-D).

#### 3.2.2. Socket Sealing in Molars

In this treatment group, tooth extraction and the post-operative course were also uneventful (Figure 2D-A).

Comparable to the findings for premolars, the process of ridge sealing at 7–10 post-operative days for solid PRF-treated molar extraction sites seemed much faster than the corresponding control group (Figure 2D). Moreover, the wound edges appeared to have been using the solid PRF as a guide to horizontally seal the ridge (Figure 2D-B,D-C). Within 7–10 days, the ridge was more or less completely sealed, and the buccal and lingual soft tissue flap edges were in a much closer distance to each other, in comparison to the corresponding findings of the control group (Figure 2D-C). At day 90, after extraction, a homogenously re-epithelialized tissue with no detectable contraction was observable on the ridge (Figure 2D-D). Consequently, almost no loss of the buccal to lingual width of the former socket walls was detectable, resulting in a ridge shape with a more prominent horizontal dimension, when compared to the findings in molars of the control group (Figure 2D-D).

### 3.3. Quantitative Assessment of Socket Sealing Process within the Study Groups

The quantitative analysis for both premolars and molar experimental groups will be presented separately. The focus is on the measurable dynamic of the socket sealing process between the corresponding groups, i.e., premolars and molars with and without PRF at the same study time points as well as on the consecutive time periods of the study within 7–10 days after extraction.

#### 3.3.1. Quantitative Assessment of the Dynamic of the Socket Sealing for Premolars of Both Groups Focused on the Same Time Points

**Figure 3:**
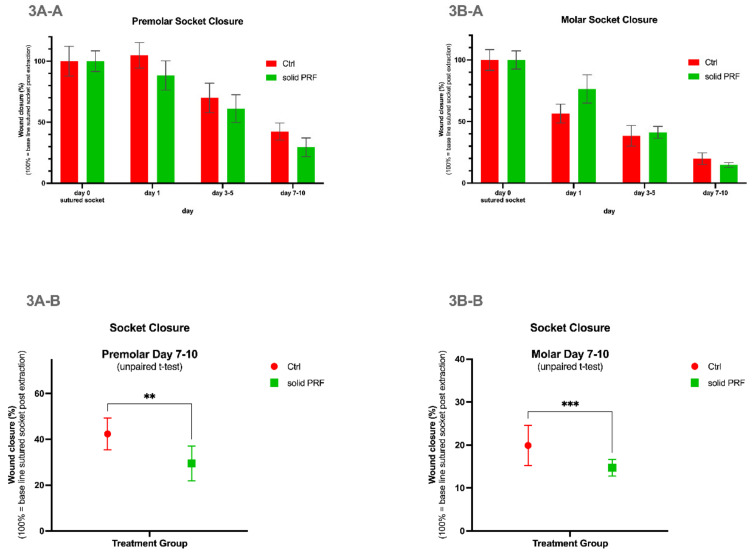
**Wound healing—quantitative socket closure evaluation.** Soft tissue wound closure was evaluated in premolars (**3A**), and molars (**3B**) up to day 7–10 post tooth extraction. Wound closure was continuously progressing over time in both control and solid PRF-treatment groups for premolars (**3A-A**) and molars (**3B-A**). At the final evaluation time point (7–10 days post extraction), the remaining wound size was statistically significantly larger in the control patient group as compared to patients in solid PRF treatment group in both premolars (**3A-B**) and in molars (**3B-B**). Data are represented as mean ± SEM for wound healing progression (**3A-A**,**3B-A**), and mean ± SD for residual wound size at days 7–10. Significance levels are as follows: “**” *p* < 0.01; “***” *p* < 0.001.

The quantitative analysis of the outer wound area sealing in premolars demonstrated that in both groups, a similar tendency of wound area reduction could be observed within the time period of 7–10 days (Figure 3A-A). The data demonstrate that wound reduction starts to become obviously measurable between day 1 and day 3–5 after operation, while no area reduction seems to be measurable within the first 24 h after extraction (Figure 3A-A). The PRF group seemed to contribute to a faster sealing process. However, there was no statistically significant difference when comparing the values of the corresponding time points of each group up to 10 days post-extraction (Figure 3A-A).

#### 3.3.2. Quantitative Assessment of the Dynamic of the Socket Sealing within Premolars of Both Groups Focused on Consecutive Time Points of the Study

The quantitative analysis of premolar wound areas for consecutive time points prior to 7–10 days revealed there was no statistically significant difference between the control and solid PRF-treatment groups. However, at 7–10 days, the solid PRF-treated group demonstrated statistically significant more sealing. Accordingly, at this time point, significantly more wound area was sealed in the solid PRF group in comparison to the control group (Figure 3A-B). This demonstrates a faster sealing process for the solid PRF-treated premolar extraction sites.

#### 3.3.3. Quantitative Assessment of the Dynamic of the Socket Sealing for Molars of Both groups Focused on the Same Time Points of the Study

The quantitative analysis of molar wound area sealing demonstrated that there was a similar tendency of area reduction in both groups within the time period of 7–10 days. The data revealed that this area reduction becomes measurable within the first 24 h following tooth extraction and continues progressing (Figure 3B-A). The solid PRF-treated group seemed to contribute to a faster sealing process. However, there was no statistically significant difference when comparing the values of the corresponding time points of each group up to 10 days post tooth extraction (Figure 3B-A).

#### 3.3.4. Quantitative Assessment of the Dynamic of the Socket Sealing for Molars of Both Groups Focused on Consecutive Time Points of the Study

The quantitative analysis of molar wound areas for consecutive time points prior to 7–10 days revealed there was no statistically significant difference between control and solid PRF-treatment groups. However, at 7–10 days, the solid PRF-treated group demonstrated statistically significant more sealing (Figure 3B-B). Accordingly, at this time point, significantly more wound area was sealed in the solid PRF group in comparison to the control group. This demonstrates a faster sealing process for solid PRF-treated molar extraction sites.

### 3.4. Quantitative Measurement of Pain in Relation to the Dynamic of Socket Sealing within the Study Groups

Data analysis of the patient completed VAS will be provided for premolars and molars separately.

#### 3.4.1. Quantitative Measurement of Pain in Relation to the Dynamic of Socket Sealing in Premolars

**Figure 4:**
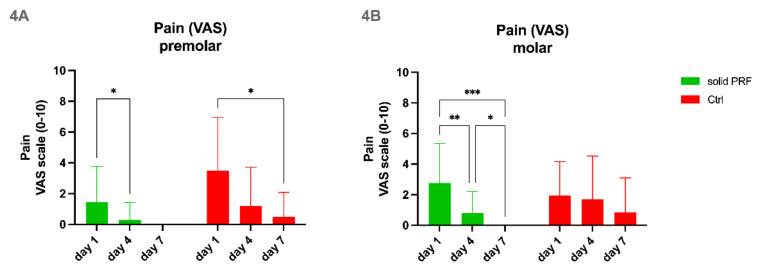
**Pain perception (VAS).** Pain perception was evaluated via visual analogue scale (VAS 0–10) in premolars (**4A**), and molars (**4B**) up to 7–10 days post tooth extraction. A continuous decrease in pain was observed for both solid PRF-treatment and control groups in both premolars and molars. A statistically significant reduction of pain from day 1 to day 4 could be observed in patients of solid PRF treatment groups for both premolars and molars. No statistically significant difference could be observed between the control and treatment group for either time point. Data are represented as mean ± SD. Significance levels are as follows: “*” *p* < 0.05; “**” *p* < 0.01; “***” *p* < 0.001.

The subjective pain analysis via VAS-scale analysis for premolars revealed that both control and solid PRF-treated groups, demonstrated a statistically significant reduction of pain over time within 7–10 days after tooth extraction (Figure 4A). It appears that solid PRF-treated patients had less pain when compared to the control group (Figure 4A). However, there was no statistically significant difference between the control and solid PRF-treated groups (Figure 4A).

#### 3.4.2. Quantitative Measurement of Pain in Relation to the Dynamic of Socket Sealing in Molars

The subjective pain analysis via VAS-scale analysis for molars revealed that only solid PRF-treated patients experienced a statistically significant reduction of pain when looking at the days 1, 4, and 7 (Figure 4B). Control group patients had a continuous reduction of pain on days 1, 4, and 7, but it was not a statistically significant reduction. Moreover, it seemed that solid PRF-treated patients had less pain, when compared to the control group (Figure 4B). However, there was no statistically significant difference in pain experienced between the control and treatment groups (Figure 4B).

## 4. Discussion

In recent years, the application of PRF in oral surgery has gained more attraction. Its autologous nature, together with its preparation without any external chemical additives, makes this blood concentrate a useful tool for oral surgical procedures. In fact, PRF can be generated in two different forms, i.e., liquid or solid. This capacity further increases the indications of PRF in various surgical procedures for oral hard and soft tissue procedures.

In the present study, the focus was on: (i) the potential capacity of solid PRF to influence the dynamic of ridge sealing after tooth extraction and (ii) the subjective evaluation of pain. To the best of our knowledge, this is the first study to analyze the effect of PRF on soft tissue wound healing and pain perception for this indication in a systematic approach. The study used quantitative image analysis of socket closure instead of a qualitative evaluation via scoring, and further observation of soft tissue healing for up to 3 months.

When looking at the dynamic of ridge sealing, it becomes apparent that the fibrin within the PRF-clot contributes in premolars and molars as a basis, upon which the de novo soft tissue can grow to seal the ridge. The solid PRF serves as a scaffold. Moreover, it has to be considered that the PRF-clot provides a scaffold rich in concentrated inflammatory cells and growth factors from the peripheral blood. Accordingly, a PRF-clot, which harbors the aforementioned agents, serves as a drug delivery system with mechanical as well as chemical properties for allowing the soft tissue to seal the surgically compromised ridge faster. Former studies could underline the presence of different inflammatory cells and growth factors within PRF, which favors soft tissue regeneration [19,30,32,33]. Further, even using PRF prepared with different protocols, the better part of published studies dealing with soft tissue healing post tooth extraction describe either significant [21,22,23,24,25] effects due to PRF treatment, or at least tendencies [26,27,28] to improved soft tissue regeneration.

When looking deeper at the process of PRF-related ridge sealing, one must understand that every tooth extraction results in a bone and soft tissue wound. The soft tissues once proximal to the extracted tooth represents the soft tissue wound, while the inner surface of the socket presents as the bone wound. Looking from this perspective, it becomes obvious that this compound wound is determined to heal via a secondary wound healing process. The root of the tooth had previously filled the socket and by its presence preserved the buccal to lingual width of the alveolar bone. The process of ridge sealing observed with the control group underlines that the wound surfaces need to orchestrate the initiation of an inflammatory response, which is known to take place in phases [34,35]. The inflammatory cells must leave the microcirculatory segments of the wound’s surrounding tissues [35], in order to reach the wound’s surface. Once these inflammatory cells have reached the wound’s surface via diapedesis [35,36], the initiation of further cell recruitment from the peripheral blood as well as the proximal tissues begins [36,37]. Thus, the wound healing process for the extraction site requires that the wound related inflammatory cells activate local fibroblasts to produce fibrous tissue to cover the inner wound surfaces via secondary wound healing. This process can take time [37]. It is known that in secondary wound healing, wound closure takes place at approximately 1 mm per day from each wound edge [38]. The data collected from the present study’s control group underlines this understanding, as in the period of 7–10 days after extraction an open wound ground could still be observed. This again reflects that the physiological wound healing process can take time until complete epithelialization is achieved [39]. In this time, the sequence involves fibrous tissue first covering the wound’s bone surface followed by epithelization of the wound. This explains the observed cone-like pattern of the internal socket surface observed in the control group. Consequently, in this time the socket wound is susceptible to infection. This might provide the explanation for the development of alveolitis sicca, which can be a post-extraction complication that must be resolved by the clinician [40].

Therefore, PRF can be considered as an agent that guides ridge sealing by presenting the socket surrounding the wound edges as a continuous allocation of mechanical as well chemical agents (i.e., concentrated fibrin and inflammatory cells with their growth factors). This allocation might be promoting the activation of fibroblasts. Accordingly, the latter cells produce a faster formation of fibrous tissue when compared with the corresponding cellular response in the wound healing process of the control group. The contribution of PRF in guiding the open healing of the ridge reflects its capacity for a faster activation of the wound healing cascade. The latter could be clearly demonstrated when quantitatively comparing the dynamic of wound closure within 7–10 days of both study groups. Here, the ridge seemed to seal faster when compared to the control group. This phenomenon might be the reason for less soft tissue contraction of the PRF group, when compared to the control group at day 90 after tooth extraction. The hypothesis here is: the more agents promoting activation of fibroblasts, the less dependent will the healing process be on myofibroblast phenotype activation [37]. In other words, wound edges reach each other faster in the premolar and molar PRF-treated groups than in the control groups. Once the bony wound is covered by a fibrous connective tissue, the epithelial cells can follow and produce an epithelial layer. This explanation is based on the findings in PRF-treated premolars and molars when compared with molars of the control group.

One way to justify the wound healing promoting capacity of PRF is to reflect about the effect of the accumulated cells and growth factory within solid PRF as a result of the centrifugation. Accordingly, within the PRF group, the wound healing cascade can start already after PRF implantation into the socket, by allowing PRF to be a natural drug delivery system to release wound healing promoting agents to the neighboring bone and soft tissue. This is due to the above-mentioned capacity of solid PRF acting as a mechanical and humoral capacity in promoting wound healing. Thus, it becomes obvious that this drug delivery dimension of PRF allows the wound healing phases run in parallel in contrast to that observed in the control group. This hypothesis is supported by the fact that in the PRF group, there is significantly faster ridge sealing at day 7–10 when compared to the control group.

In this study, another focus was on patients’ subjective pain perception, which was analyzed by means of a VAS provided to patients following their surgery. The data demonstrated that there is no significant difference observed when comparing the results of the PRF-treated and control groups.

Some previous studies reported in the literature concluded that the use of PRF in socket preservation lead to significantly less postoperative pain [22,23,26]. However, other studies found no significant effects on pain perception with PRF treatment, but did conclude that there was a tendency for less postoperative pain [21,27,29]. Even though there was no statistically significant effect on pain in this study, tendencies to improve pain management could be detected here as well. It seemed that the patients of the PRF group had less intense or total pain in comparison to that of the control group. Consequently, the pain might be related to the longer time necessary for ridge sealing within the control group in comparison to the PRF group.

Based on this data, it can be concluded that the application of PRF had no direct effect on pain perception by the patients. Therefore, PRF should be considered as an agent guiding ridge sealing rather than a pharmaceutical agent interfering with neuronal processes responsible for pain. There are multiple studies that seemed to have observed less pain in PRF treated patients [22,23,26]. However, the data of the present study cannot support those findings. Ongoing studies by the authors are examining the extent of the positive effect PRF treatment has on the secondary wound healing process and possible bone maturation within the extraction socket.

## 5. Conclusions

The present study analyzed the effect of solid PRF-treatment on the wound healing process following the extraction in premolars and molars. The evaluation methods assessed the ridge sealing response in a prospective, parallel-arm, clinical, single blind, randomized controlled trial (RCT). Solid PRF-treatment of premolar and molar extraction sockets was demonstrated to have a positive influence on the acceleration of secondary wound healing cascades. The solid PRF-treatment appears to predictably guide open wound healing in both premolar and molar extraction sites with less contraction of the soft tissue in comparison to the control group, i.e., non-PRF treated group. However, for pain perception there was no significant difference observed between treatment and control groups. Further data will be necessary to identify the role less expressed that the contraction of the soft tissue has on subsequent: (i) bone formation within the socket, (ii) long-term dental implant stability, and (iii) occurrence of peri-implantitis.

## Data Availability

No new data were created or analyzed in this study. Data sharing is not applicable to this article.
